# The role of drug regulatory authorities and health technology assessment agencies in shaping incentives for antibiotic R&D: a qualitative study

**DOI:** 10.1186/s40545-023-00556-x

**Published:** 2023-03-27

**Authors:** Cecilia Kållberg, Liv Mathiesen, Unni Gopinathan, Hege Salvesen Blix

**Affiliations:** 1 Institute of Health and Society, Faculty of Medicine, University of Oslo, Forskningsveien 3A, 0373 Oslo, Norway; 2 Norwegian Institute of Public Health, Lovisenberggata 8, 0456 Oslo, Norway; 3 School of Pharmacy, University of Oslo, Sem Sælands Vei 3, 0371 Oslo, Norway

**Keywords:** Regulatory authorities, Marketing authorization, HTA, Antibiotics, Antibiotic R&D, Novelty, Public health need, Unmet medical need

## Abstract

**Background:**

Few antibiotics have entered the market in recent years despite the need for new treatment options. Some of the challenges of bringing new antibiotics to market are linked to the marketing authorization and health technology assessment (HTA) processes. Research shows great variation in geographic availability of new antibiotics, suggesting that market introduction of new antibiotics is unpredictable. We aimed to investigate regulatory authorities’ and HTA agencies’ role in developing non-financial incentives to stimulate antibiotic research and development (R&D).

**Methods:**

We conducted individual, semi-structured, stakeholder interviews. Participants were recruited from regulatory authorities (EMA and FDA) and HTA agencies in Europe. Participants had to be experienced with assessment of antibiotics. The data were analyzed using a deductive and inductive approach to develop codes and identify key themes. Data were analyzed using thematic analysis including the constant comparison method to define concepts, and rival thinking to identify alternative explanations.

**Results:**

We found that (1) interpretation of key concepts guiding the understanding of what type of antibiotics are needed vary (2) lack of a shared approach on how to deal with limited clinical data in the marketing authorization and HTA processes is causing barriers to getting new antibiotics to market (3) necessary adaptations to the marketing authorization process causes uncertainties that transmit to other key stakeholders involved in delivering antibiotics to patients.

**Conclusions:**

A shared understanding of limited clinical data and how to deal with this issue is needed amongst stakeholders involved in antibiotic R&D, marketing authorization, and market introduction to ensure antibiotics reach the market before resistance levels are out of control. Regulatory authorities and HTA agencies could play an active role in aligning the view of what constitutes an unmet medical need, and direct new economic models towards stimulating greater diversity in the antibiotic armamentarium.

**Supplementary Information:**

The online version contains supplementary material available at 10.1186/s40545-023-00556-x.

## Background

Antibiotic resistance has increased the need for new antibiotics to combat bacteria that no longer respond to treatment, but bringing new drugs to market has proven difficult [1]. Antibiotic treatment courses usually have a short duration, are scientifically challenging to develop, and as resistance develops, risk becoming ineffective [2–4]. The current economic model directly links return of investment to unit sales, and incentivizes companies to maximize sales [5]. At the same time, interventions are put in place to reduce the use of antibiotics to slow down antibiotic resistance. Ultimately, the financial return on developing new antibiotics is considered too low by the pharmaceutical industry, and many companies have exited the field [3]. To attract companies to reinvest in antibiotic R&D new financial and non-financial incentives are needed [3–7].

Some of the non-financial incentives considered to influence innovation are linked to the process of marketing authorization, as well as pricing and public reimbursement. Designing clinical trial programs have proven difficult, both due to scientific challenges as well as differences between countries and regions regarding the regulatory requirements on the design of clinical trials included in the marketing authorization application for new antibiotics [8–10]. Regulatory authorities have sought to address these challenges by strengthening the collaboration between them as well as with industry, and by developing guidelines for antibiotics addressing areas of important need [11–19]. Regulatory authorities have a high concordance when approving antibiotics and have become more harmonized with respect to approval time, but differences are still seen concerning target indications and decisions to conduct priority review/accelerated assessment [20–28].

After marketing authorization, pricing and reimbursement schemes are decided through national processes that, among others, involve the use of health technology assessments (HTA). HTAs are generally understood as multidisciplinary, systematic and transparent processes for determining the value (e.g., cost-effectiveness) of a health technology [29], and are used by governments and public and private health insurance schemes to inform reimbursement decisions. In contrast to how other drugs are evaluated, there are specific challenges that are unique to antibiotics [30]. This includes measuring the public health value of antibiotics, since antibiotics not only helps the patient receiving the medication but prevents the infection from spreading to other people, as well as enabling other types of treatments and procedures, such as chemotherapy and surgery. Use of HTA to inform reimbursement decisions is increasing and represent a financial factor that is likely to influence innovation of antibiotics [31–35], yet little is known about HTA agencies response to the challenges linked to making new effective antibiotics available.

Research shows a great variation in geographic availability and considerable lag-time between regions in the uptake of new antibiotics [36]. This suggests that there are challenges in the marketing authorization and HTA processes that makes market introduction of new antibiotics unpredictable. This could create uncertainty among innovators about how their product will be valued by healthcare systems. While most of the literature pertaining to incentivizing antibiotic R&D has focused on the role of new financial incentives and payment models, there is an increasing interest in regulatory authorities and HTA agencies role in stimulating innovation of antibiotics targeting unmet needs, including resistant bacteria [12, 26, 35, 37–39].

This study aimed to answer the question if and in what way regulatory authorities and HTA agencies can contribute to the attempts to stimulate antibiotic R&D, primarily through non-financial incentives. We investigate this question by exploring the collective experiences with assessing and valuing antibiotics and explore the perceptions of those working in regulatory authorities and HTA agencies. We identify barriers and facilitators to antibiotic R&D and market introduction unique to the processes managed by regulatory authorities and HTA agencies, and potential barriers and facilitators resulting from the interaction between these two. Finally, we explore potential strategies for getting new antibiotics to market.

## Methods

### Study design

To answer the research question, this study used a qualitative study design involving semi-structured interviews with experts from regulatory authorities and national HTA agencies. The COREQ checklist was used to ensure explicit and comprehensive reporting of study design characteristics (Additional file 1) [40]. The design of the study, development of the interview guide, recruitment strategy, and analysis of results were informed by a document review exploring the use of the terms unmet medical need, novelty/novel drugs and innovation by EMA, FDA and WHO (Table 1), a literature review and previous research by the research team on the market introduction of new antibiotics [36]. The purpose of the literature review was to determine the primary theoretical perspectives guiding interpretation of the qualitative data and was conducted for published articles on PubMed and Google scholar using combinations of the following search terms: “regulatory approval”, “marketing authorization”, “market introduction”, “HTA”, “antibiotics”, “antimicrobials”, generic and original names of antibiotics approved during the past 20 years. Previous research by the research team included analysis of sales data and a document review of EMA and FDA approval documents and summaries of product characteristics (SPCs) of new antibiotics approved between 1999 and 2014 [36, 41, 42].

**Table 1:** Definitions and use of key concepts in major normative institutions

	Innovation	Novelty	Unmet medical need
WHO	Defined as "Absence of cross-resistance to existing antibiotics, new chemical class, new target, or new mechanism of action."	Not defined	Not defined
EMA	Innovative medicine is defined as “A medicine that contains an active substance or combination of active substances that has not been authorised before."	Novel drugs/therapies/treatments are frequently referred to but not defined for human medicines. The focus is on whether a new antibacterial agent belongs to a new class that has a unique mechanism of action and, therefore, has the potential to address an unmet medical need. For veterinary medicines, novel therapies are defined as "therapies entirely new to veterinary medicine either, because they are genuinely novel and have not been previously used in the context of a medicine, or new only to the veterinary domain, although well-known in terms of research, and possibly in the context of human medicine."	Defined as “a condition for which there exists no satisfactory method of diagnosis, prevention, or treatment in the Union or, even if such a method exists, in relation to which the medicinal product concerned will be of major therapeutic advantage to those affected”. EMA also state that "target organisms expected to respond to treatment with the new agent and for which there are few remaining treatment options" can be considered an unmet medical need
FDA	Innovative drugs are not clearly defined but referred to as " new treatment options for patients and advances in health care for the American public."	Novel drugs/novelty is often referred to but not clearly defined. Novelty is partly addressed using the term New Molecular Entity (NME). This concept refers to an active ingredient that does not contain any active moiety previously approved under section 505 of the Federal Food, Drug, and Cosmetic Act nor previously marketed as a drug in the United States. However, FDA uses the term “novel drug” in alternative pathways designed to facilitate faster drug approval. A “novel drug approval” is considered Accelerated Approval if the drug treats a serious condition and offers a significant improvement over existing therapies. The improvement can be demonstrated by an effect on either a surrogate endpoint that is likely to predict clinical benefit or on a clinical endpoint that can be measured earlier than irreversible morbidity and mortality or is reasonably likely to predict an effect on irreversible morbidity and mortality or another clinical benefit (i.e., an intermediate clinical endpoint). Similarly, a “novel drug marketing application” receives a “Fast Track” status when the drug is intended for treating a severe or life-threatening illness or medical condition, and it shows potential for fulfilling unmet medical needs in this area. In addition, a drug may also receive Fast Track status if it is designated as a qualified infectious disease product, which is a concept under a FDA scheme to incentivize the development of antibacterial and antifungal drugs to treat serious or life-threatening infections	Defined as "a condition whose treatment or diagnosis is not addressed adequately by available therapy. An unmet medical need includes an immediate need for a defined population (i.e., to treat a serious condition with no or limited treatment) or a longer-term need for society (e.g., to address the development of resistance to antibacterial drugs).”

Definitions of innovation, novelty, and unmet medical need by WHO, EMA and FDA [13–15, 17, 19, 43, 45–52]

### Theoretical perspectives—the concepts of novelty and unmet medical need

To mitigate the impact of resistance it is desirable that new antibiotics work in different ways than already marketed antibiotics. WHO defines innovation in antibiotic R&D as no cross-resistance to existing antibiotics, new chemical class, new target, or new mechanism of action [43]. In addition, WHO has listed the pathogens that pose the greatest threat to public health and, therefore, should be prioritized in antibiotic R&D [44]. These pathogens were identified using a multicriteria decision analysis including the following criteria: mortality, health-care burden, community burden, prevalence of resistance, 10-year trend of resistance, transmissibility, preventability in the community setting, preventability in the health-care setting, treatability, and state of the pipeline [44]. The EMA and FDA have developed pathways to enable the approval of new antibiotics targeting serious or life-threatening infections and unmet medical needs, including the use of limited clinical data. In this work, EMA and FDA refer to, but do not always define, innovation, novelty/novel drugs, and unmet medical need [13–15, 17, 19, 45–52]. Table 1 provides a summary of how these concepts were defined and discussed by EMA, FDA and WHO. Key insight from the literature review was that stakeholders involved in drug R&D and market introduction including regulatory authorities, HTA agencies, patients, payers, and industry use multiple criteria to assess unmet medical need [37, 53]. These can be divided into three categories: adequacy of alternative treatments, disease burden, and population size, but with no alignment on how to measure these criteria [37, 53]. Ultimately, unmet medical need and novelty, which reflect the value of an antibiotic, represent broad, unspecific terms. When analyzing the qualitative data, the key concepts novelty and unmet medical need guided our attention. Specifically, we examined how interviewees from regulatory authorities and HTA agencies interpreted these concepts and described their application in assessing and evaluating new antibiotics. Our analysis aimed to gain a deeper understanding of how these key concepts were used in the context of antibiotic development and approval processes. We paid close attention to the nuances of interviewees' descriptions, as well as any commonalities or divergences in their interpretations. By doing so, we sought to identify patterns and insights that could inform future efforts to improve antibiotic development and regulation.

### Interview guide

We developed a semi-structured interview guide with open-ended questions (Additional file 2). The interview guide consisted of 17 questions sorted under five main topics, informed by the theoretical perspectives described above: (1) factors of importance when antibiotics are evaluated: (2) changes in the perspective of what an antibiotic is: (3) perception of novelty and need: (4) barriers to get novel antibiotics to market: and (5) interventions to improve antibiotics R&D, assessment and approval to assure effective antibiotics reach the market. The interview guide was evaluated and adjusted after a pilot interview. Adaptations were made to the guide to assure that the questions were relevant for the participants depending on which stakeholder group they represented.

### Study recruitment and interviews

Participants for semi-structured interviews were recruited from regulatory authorities (EMA and FDA), and HTA agencies in Europe. Participants had to be experienced with assessment of antibiotics and work in institutions that held responsibility for marketing authorization and assessment of benefit/risk or assessments of value. Individuals were identified through regulatory networks and internet searches of relevant agencies and contacted using emails presenting the research team, background and aim of the study. A total of 46 emails directed at agencies assessing drugs for national use (including HTA) and 36 emails directed at medical agencies linked to the EMA network were sent to recruit participants fulfilling the criteria stated above. Participants were asked to recommend relevant candidates for additional interviews to enable snowball sampling [54]. Suggested individuals were discussed within the research team and added if perceived valuable for the data collection process. Written or oral consent to participate in the study, including whether the participant was comfortable with the interview being audiotaped, was obtained before the interview.

Interviews were conducted over telephone or skype, between December 2017 and June 2019, and were audiotaped. The median length of the interviews was 86 min (52–151 min). Participants received an overview of the topics to be discussed prior to the interview, and each interview began with an introduction of the study and primary investigator CK. CK conducted and led the primary analysis of the interviews, which were transcribed and anonymized before analysis. Participants were contacted by email if clarifications were needed after the interview. Several different techniques were used to strengthen trustworthiness and rigor, including a secondary independent analysis of the interviews by HSB and LM, and two consensus sessions, where CK, HSB and LM discussed the findings. Saturation was determined after constantly comparing experiences and responses of the participants against each other and by appraising the richness of experiences shared by participants [55]. We used deductive and inductive reasoning when conducting thematic analysis and developed themes explaining organizational processes related to the assessment and approval of new antibiotics. We analyzed the qualitative data applying the five-cycle phase described by Yin [54], which includes: (1) compiling, (2) disassembling, (3) reassembling, (4) interpreting, and (5) concluding. An iterative strategy was used, moving between steps (2)–(4) to allow for a continuous re-assessment of analytical codes, concepts, and themes. Our application of thematic analysis [56] involved the constant comparison method known from grounded theory to define concepts, rival thinking to identify alternative explanations for the observed patterns in the data [57], and identification of divergent views to challenge generalization [58]. The coding of qualitative data was done in NVivo 12 [59]. Findings were shared with respondents for commenting and validation and to strengthen trustworthiness of the findings.

## Results

### Overview

The study included 19 participants. One interview included three participants and another two participants from the same agency. Three participants provided written responses. Together the participants represented 11 countries in Northern, Western, Southern and Central Europe, and the US. The participants representing regulatory authorities had a background in clinical medicine, biology, or pharmacology, while representatives from HTA agencies had a background in clinical medicine, pharmacology, and health economics. One participant had previous experience working in pharmaceutical industry. The participants had two to over 20-years experience working with evaluation of antibiotics. Participants working with marketing authorization in the EMA were often linked to national agencies, either as an employee and working as a rapporteur/co-rapporteur for the EMA, or by having worked for the national agency before transferring to the EMA. Moreover, several HTA agencies that were approached in the recruitment process responded that they so far had no experience in assessing new antibiotics. Sixteen of the 19 participants, therefore, had experience working with marketing authorization as well as HTA, or only with marketing authorization, while three participants had solely worked with HTA of antibiotics. The thematic analysis comprised five themes: (1) uncertainties and disagreements regarding to what extent the marketing authorization process can and should be adapted; (2) increased risk of irresponsible use of antibiotics; (3) the increasing population-level spread of resistant strains means that benefit/risk assessment of new antibiotics will vary over time; (4) adjustments to regulatory requirements for antibiotics misaligned with evidence needs for determining value with HTAs; and (5) different interpretations of “novelty” and “need” translates to differing views on what kinds of antibiotics are urgently needed, see Table 2 for major themes and corresponding qualitative codes. Overall, participants agreed that the main barrier getting new antibiotics to market is lack of economic incentives and did not consider the marketing authorization or HTA processes as major barriers to get new antibiotics to market.

**Table 2:** Major themes and corresponding qualitative codes

Themes	Level 2 codes	Level 1 codes
Uncertainties and disagreements regarding to what extent the marketing authorization process can and should be adapted	Current challenges for regulatory authorities are caused by AMR	Regulatory authorities have addressed most structural challenges in marketing authorization
		Current challenges in marketing authorization are due to lack of clinical data
	Adaptation to the marketing authorization process causes new challenges	Increased importance of PK/PD
		Uncertainties of how to use PK/PD data
		HTA agencies asking for more clinical data
	Balance between addressing AMR and upholding good quality	Continuous work to address the future antibiotic need, need to find new ways to meet new challenges
		Unacceptable to reduce requirements in the marketing authorization process to address AMR
		Regulatory authorities have adapted as much as they can
Increased risk of irresponsible use of antibiotics	Lack of clinical data makes it difficult for physicians to make an informed decision and uphold responsible use	Company holds power over how a drug is used, not forced to consider AMR
		Limited clinical data lead to limited post-approval data
		Limited clinical data lead to greater uncertainty regarding the benefit/risk profile of the drug
	Adhering to the regulatory authorities’ recommendations can both reduce and increase the risk for irresponsible use	Antibiotics approved with limited clinical data risks incentivizing off-lable use
		Regulatory authorities clear on circumstances for approval and intended use
	Limited jurisdiction and clear stakeholder perception	Companies and clinicians have the final say in how an antibiotic is used, not under the control of regulatory authorities and HTA agencies
The increasing population-level spread of resistant strains means that benefit/risk assessment of new antibiotics will vary over time	Marketing authorization based on limited clinical data results in increased safety risks	Lack of clinical data leads to uncertainties regarding benefit/risk profile
		Smaller safety databases lead to uncertainties regarding the safety profile
		Using an antibiotic in a limited population leads to limited post-approval data
	Accepting a higher level of uncertainties regarding the safety profile, if benefits overweigh potential safety risks, does not mean there is an increased acceptance for safety risks	Benefit and risk are relative to the situation
	Lack of effective antibiotics targeting unmet needs, including AMR, is a threat to public health	Delay in antibiotic R&D, need to act now before resistant bacteria have spread too much
		Continuous work to address the future antibiotic need, need to find new ways to meet new challenges
Adjustments to regulatory requirements for antibiotics misaligned with evidence needs for determining value with HTAs	Regulatory authorities actively work to address AMR	Regulatory authorities have adapted as much as they can
	Different objectives	HTA agencies assess cost-effectiveness, regulatory authorities assess benefit/risk
	Regulatory authorities consider it problematic that HTA agencies demands additional clinical data in cases, where this is difficult to obtain	HTA agencies ask for different data than regulatory authorities
		HTA agencies ask for proof of superiority
		If the demand for clinical data is too high companies will exit the field of antibiotic R&D
	Disagreement between regulatory authorities and HTA agencies whether HTAs are causing barriers to get new antibiotics to market	Targeting an unmet medical need increases the HTA-score
		HTA does not look at public health impact of a resistant bacteria
		Antibiotic prices are too low
	Limited experience with HTAs of antibiotics	Few countries conducting HTAs
		Few new antibiotics have reached the market in recent years resulting in limited experience conducting HTAs for new antibiotics
Different interpretations of ‘novelty’ and ‘need’ translates to differing views on what kinds of antibiotics are urgently needed	Need for a broad armamentarium	Need for antibiotics to treat hospitalized patients suffering from infections caused by multidrug resistant bacteria
		Need for new small-spectrum antibiotics to reduce development of AMR in the population at large
	Novelty has multiple meanings	Drugs can be novel based on its own characteristics or based on the circumstances of the situation it is being used in
	Unmet need is a dynamic term	Different needs in different regions
		Size of patient groups
		Unclear when additional antibiotics in the same class no longer adds value

Level 1 codes, level 2 codes and themes identified during data analysis

### Main findings

1. Uncertainties and disagreements regarding to what extent the marketing authorization process can and should be adapted

Participants from regulatory authorities described that past challenges in marketing authorization of antibiotics, such as authorities asking for site-specific studies, different endpoints and non-inferiority margins, had largely been addressed. The challenges antibiotic resistance creates for evidence generation and assessment of new antibiotics was now the main concern. Interviewees highlighted the difficulty recruiting enough patients in clinical trials due to low prevalence of target pathogens and assessing the submitted documentation when based on scarce clinical data. In response to this challenge, new guidelines highlighting the use of pharmacokinetic/pharmacodynamic (PK/PD) data has been introduced as a main strategy for addressing limited clinical trial data.

Interviewees considered these adaptations a necessity; however, some expressed concerns over their use: “the lack of PK/PD markers is becoming more and more an issue”, “PK/PD targets are not that well defined “and “…many [assessors] are uncomfortable using PK/PD data…”. With respect to how much regulatory authorities can adapt to further facilitate marketing authorization of antibiotics, interviewees expressed views across the spectrum. Most interviewees expressed that regulatory authorities have reached a point, where they cannot make further adjustments and still uphold a good standard. In contrast, one interviewee pointed out that adaptations to the authorization systems will have to continue to meet the need for new antibiotics “I think we will have to become more creative when it comes to what expectations you have on the clinical side”. One participant suggested the use of alternative data sources to compensate for limited clinical trial data, “most countries have nowadays electronic records, we can transfer data from electronic patient records to clinical trial files”. At the other end of the spectrum another interviewee questioned adapting the regulatory process “You can say that you don't ask for anything irrational, but I think it is a totally wrong concept that you should reduce requirements to get more product in one or another field”.

2. Increased risk of irresponsible use of antibiotics

A second challenge, related to approving antibiotics based on limited data, was the risk that the antibiotic will be used irresponsibly once it enters the market. New antibiotics, targeting an unmet need, should only be used when there are no other options to avoid development of resistance but also given the limited availability of safety data. For example, one interviewee voiced a concern that companies might attempt to get a product approved for an indication considered an unmet medical need, but then encourage off-label use for other susceptible infections with higher prevalence to be able to generate return on investment. “The risk is that these drugs might also be very effective for treating the more regular UTI’s or skin infections …and will be used in the field that way, because there has been a publication or has been a study that says it has been effective in this indication …whilst we don’t have the data to support that”. However, another participant commented that such alternative use may simply reflect reasonable clinical judgement: “the use of a new drug depends on multiple factors including level of resistance and patient population, so the final use of the drug can differ from what was intended, and this might not be wrong but simply mirror the development of the situation… clinicians will use what they need.”

3. The increasing population-level spread of resistant strains means that benefit/risk assessment of new antibiotics will vary over time

Interviewees expressed different views about whether antibiotics approved with limited clinical data presented an increased safety risk. One group of participants held the view that approval under such circumstances entails accepting an increased safety risk. This view was based on two arguments. First, that this would lead to safety issues going unnoticed prior to marketing authorization. Second, that these safety issues will likely continue to go unnoticed post-approval, given that new antibiotics will be kept as a last-line treatment option and that evidence about these drugs would take time to accumulate. In contrast, another group of interviewees argued that if resistant strains continue to reduce antibiotic effectiveness, and if treatment options for these strains continue to be lacking, it would be possible for a new antibiotic to have a positive benefit/risk profile even if this comes with additional safety risks compared to existing therapy. As one participant stated “benefit/risk is not an equation, it is a judgement.” Another interviewee reflected “if you wait to conduct clinical trials until you have a population that is big enough you’ve waited too long. You’ve allowed the resistant strain to spread… It is important to not make it too difficult.” Accordingly, marketing authorization of an antibiotic at a point in time when spread is limited may increase the risk of adverse events for individual patients, while marketing authorization of an antibiotic at a point in time when clinical data is available increases the risk for the population at large in the form of pandemics. Participants representing regulatory authorities emphasised that approval based on limited clinical data is clearly communicated in the summary of product characteristics (SPC), e.g., that the antibiotic should be used only in situations where there are no other options. By following these recommendations physicians minimize safety risks. Participants representing regulatory authorities were also clear about the fact that marketing authorization will not be given unless clinical data is sufficient to assess benefit and safety risks.

4. Adjustments to regulatory requirements for antibiotics misaligned with evidence needs for determining value with HTAs

Representatives from regulatory authorities perceived that HTA in some cases risk becoming a barrier to bringing new antibiotics targeting unmet medical needs to market. Regulatory authorities are increasingly accepting limited clinical trial data in the authorization process. In contrast, HTA agencies conventionally ask for additional or different data, such as data proving superiority or data on a specific patient group, to determine the value of a new antibiotic. In the absence of these data, the product risk failing the criteria for public reimbursement and the company would be discouraged from marketing the drug. Representatives from regulatory authorities saw the need for HTA agencies to adapt to the changes implemented in the marketing authorization process, including perception of unmet need, and developing approaches to manage limited clinical data, described under the first theme.

Participants experienced with HTA acknowledged that companies could be asked to collect additional data for the HTA than what was considered satisfactory for the marketing authorization process. However, this was not considered a major problem, since a valuable antibiotic would receive a positive assessment regardless of limited clinical data, since in the case of a new antibiotic, claiming to target an unmet need, there would be no available treatment that would offer a more cost-effective alternative. Moreover, representatives from HTA agencies argued that due to health care budgets and the European pricing system, which keeps antibiotic prices relatively low, the chances for companies to generate return on investment for new antibiotics are small regardless of the value determined using HTA.

It should be noted that the experience with using HTAs for assessing the value of antibiotics was limited. Many representatives from national agencies responsible for drug assessment expressed during the recruitment process that they either do not conduct HTAs for antibiotics, that HTA is only conducted if a company requests it, or that they are in the early process of setting up HTAs. A partial explanation for this limited experience is that few new antibiotics have been developed in recent years. While regulatory authorities have developed guidelines to address antibiotic resistance, representatives from HTA agencies did not report any similar initiatives in their field. Instead, representatives from HTA agencies mentioned the need to continue the work to harmonize HTA between European countries to assure that the additional data collected by a company can inform assessment of value in multiple jurisdictions. A challenge in this process was how to choose a comparator for a new antibiotic that would work for multiple countries. Compared to other types of drugs, the different levels of antibiotic resistance across countries, as well as antibiotic treatment guidelines, present a unique challenge for harmonized post-approval evidence generation and value assessment.

5. Different interpretations of ‘novelty’ and ‘need’ translates to differing views on what kinds of antibiotics are urgently needed

‘Novelty’ and ‘ unmet medical need’ are key concepts guiding the understanding of what kind of antibiotics are needed [60]. Interviewees held different interpretations of the concepts of ‘novelty’ and ‘unmet medical need’. Most participants defined ‘novelty’ as ‘new in class’—meaning the first antibiotic in a new class. However, some argued that being ‘new in class’ is insufficient if the goal is to combat antibiotic resistance. These interviewees argued that ‘novelty’ must entail discovering antibiotics with no known resistance mechanisms against the compound. Others argued that novelty could entail discovering a new bacterial target or formulation that would create new treatment options. One participant divided ‘novelty’ into three groups: “I would describe novelty in these three areas; new mechanism of action, niche indications (targeting a specific resistant strain), and targeting indications where the available antibiotics are no longer, at least in certain geographical areas, working”.

The concept of an antibiotic targeting a public health need, often termed ‘unmet medical need’, was seen to differ between regions and over time due to differences in disease prevalence and different needs in different patient groups. Respondents found it difficult to know when an unmet need had been filled. For example, one interviewee expressed: “is it an unmet need if only a subpopulation of 50 people need the drug, what about a 1000 people?…There will always be benefit to some patients.” Another key perspective was whether to emphasize benefits to individual patients or benefits to society at large, since this has implications for which antibiotics that should be developed. One interviewee expressed: “Benefit to society, benefit to patients, who are we developing for? The great masses or patients in hospitals?” Accordingly, a globally uniform understanding of what constitutes antibiotics targeting an unmet medical need was not deemed possible.

Crucial to generating public health value is controlling the spread of antibiotic resistance. Accordingly, another interviewee expressed that “antibiotics of a public health value are antibiotics that reduce resistance, either new ones with a new mechanism of action or old, small-spectrum antibiotics”. Reflecting further, the interviewee added: “I don’t think broad-spectrum antibiotics would be the answer to the global health problem, but we need it as well.” Such an understanding implies that judicious use of antibiotics with new mechanisms of action or of narrow-spectrum antibiotics are most important to public health, and that broad-spectrum antibiotics, although being of great importance for individual patient, should be restricted given that they are strong drivers of antibiotic resistance.

Interviewees reflected on how the multiple understandings of unmet medical need could be translated to which antibiotics should be incentivized for development, and that there is a need for a broad armamentarium. It will not be enough focusing on the current gaps in antibiotic treatments; instead, there is a need for a “tool kit” to be able to stay ahead of antibiotic resistance. To this end, respondents from both regulatory authorities and HTA agencies suggested continued efforts to increase communication with companies, including scientific advice at an early stage, to guide development towards unmet medical needs. However, this was considered to have limited impact, since companies have the final say over target pathogen and indication and may choose to not comply with the advice given.

## Discussion

This study aimed to answer if and in what way regulatory authorities and HTA agencies can contribute to bringing new antibiotics to market, primarily through non-financial incentives, by examining the perspectives of regulatory authorities and HTA agencies in ongoing efforts to motivate R&D of antibiotics. We found that (1) interpretation of key concepts guiding the understanding of what type of antibiotics are needed vary (2) lack of a shared approach on how to deal with limited clinical data in the marketing authorization and HTA processes is causing barriers to getting new antibiotics to market (3) necessary adaptations to the regulatory authorization process causes uncertainties that transmit to other key stakeholders involved in delivering antibiotics to patients. However, participant did not consider themselves to be in a position to have a major impact on antibiotic R&D, given that lack of financial incentives was perceived as the major barrier. They suggested continued and increased discussion with the pharmaceutical industry to guide development towards unmet medical needs as their way of contributing to a diverse and effective antibiotic armamentarium.

Our study identified that the interpretation of key concepts guiding antibiotic R&D—'novelty’ and ‘unmet medical need’—differed widely. This is in line with other research showing that the definition of unmet medical need, used as a synonym to ‘public health need’ by our respondents, differs between stakeholders including regulatory authorities, HTA agencies, industry, payers, health care professionals and patients [37]. This varied understanding of novelty and unmet medical need may reflect the need for diversity in the antibiotic armamentarium, highlighting that it is not only the value of an individual antibiotic that needs to be taken into consideration when incentivizing antibiotic R&D, but the value generated by the entire antibiotic arsenal. Nevertheless, it creates a challenge for policy makers tasked with developing incentives to stimulate antibiotic R&D. Much attention is currently given pull incentives, which are financial payments made at the end of the R&D pipeline designed to primarily incentivize big pharmaceutical companies to reinvest in antibiotics [3–7]. Their impact will depend on a general agreement amongst decision makers on which criteria makes an antibiotic eligible for an incentive, and a design that allows the combined effect of multiple incentives to generate diversity in the armamentarium. To achieve this goal there needs to be a shared understanding between policy makers developing these financial incentives, stakeholders evaluating new antibiotics, and industry on which characteristics constitutes value in an antibiotic. Second, these incentives need to be implemented together with systems for continuous monitoring of new unmet needs and communication when needs have been met. Regulatory authorities and HTA agencies could play an important role informing this process, since their evaluations of new antibiotics heavily influence the value.

Marketing authorization of antibiotics based on limited clinical data, made possible by changes to regulatory requirements, introduces uncertainties regarding the properties of the drug as well as its value [61]. These uncertainties reside within regulatory authorities, which is expected, since the need for strategies to combat antibiotic resistance will likely be never ending and must adapt over time. However, these uncertainties also seem to transmit to key stakeholders—HTA agencies, payers, health care professionals—involved in delivering antibiotics to patients, who may not agree on the value of a particular antibiotic based on the available data. The misalignment between the regulatory authorities and HTA agencies in generating evidence to assess new antibiotics when data are limited highlights this problem. In the current situation HTA agencies have varied influence on antibiotic prices across Europe depending on the country. However, the use of HTA is increasing, efforts are under way to harmonize the HTA processes across Europe, and new economic incentives increasing the return on investment for new antibiotics are being promoted. Accordingly, the HTA process will likely play an important role in assuring funds are allocated in a way that strengthens the antibiotic armamentarium. In addition, there have been concerns that limited clinical data could lead to HTAs, and in extension pricing and reimbursement decisions, varying between countries which would undermine the development of pooled funding to finance new economic models to stimulate antibiotic R&D [61]. Therefore, to reduce misalignment between the marketing authorization and HTA processes there is a need for increased collaboration between regulatory authorities and HTA agencies on how to deal with limited clinical data and the development of a shared understanding of what constituted an unmet medical need.

Another example of how uncertainty generated from limited clinical data transmit to other stakeholder is a greater responsibility put on clinicians to manage the use of new antibiotics if data on specific patient groups is not available. In addition, these antibiotics will be considered last line treatments which puts additional responsibility on clinicians to avoid unnecessary use. This is supported by research showing that approval based on limited clinical data has been linked to increased safety risks leading to safety actions as well as increased off-label use [11, 38, 62]. Since antibiotics can be prescribed by any physician, regardless of specialization, and in some cases over the counter, the medical community needs to take an active approach to the adaptations made to the regulatory authorization process and develop strategies to assure new antibiotics are used in a safe and responsible way.

Clinical data for new antibiotics will likely continue to be difficult to generate in a timely fashion with respect to country-level reimbursement decisions. At the same time, countries will increasingly need new antibiotics in response to increasing resistance, which is why interventions to stimulate antibiotic R&D is urgently needed. Developing a shared understanding of what constitutes unmet medical need and how to measure value of new antibiotics are important features of this process [37]. A starting point for this work could be the criteria used in the development of the WHO priority pathogen list [44], or the list of factors suggested to be added to the HTA process of new antibiotics to capture the true public health value of new antibiotics [35]. Both these lists of factors aim to identify characteristics that makes the antibiotic valuable to patients and society. A selection of them could make up a shared base for evaluating a drug’s value. Regulatory authorities and HTA agencies may play a key role in aligning the view of what constitutes an unmet medical need and assure that antibiotics’ full value to health care systems is recognized in the reimbursement process [35], thereby directing new economic models to motivate diversity in the antibiotic armamentarium.

This study has a number of strengths and limitations. Our research group has experience in multiple fields including clinical medicine, drug monitoring and responsible use, public health, regulatory approval, quantitative and qualitative research which has enriched the discussions and understanding of the findings. While all members of the team have experience working in international collaborations, the authors originate from and work in the Nordics. None of the authors have experience working in the pharmaceutical industry. These factors likely influence our views of what potential barriers and facilitators to antibiotic R&D and market introduction may be. We struggled to recruit participants experienced with HTA of antibiotics. This is explained by the fact that few antibiotics have been developed in recent years, and that many countries do not conduct HTA routinely for new antibiotics. Stronger representation from participants solely working in HTA would likely have enriched the data and possibly revealed other aspects not identified in the data we gathered. However, the participants included in the study are well-experienced in antibiotic evaluation and associated with strong and solid medical agencies in Europe and the US. It would have been valuable to add regulatory authorities and HTA agencies from other countries outside of the US and Europe, but this was not possible due to limited resources. For example, inclusion of participants from PMDA in Japan could have offered an interesting aspect to our data, since antibiotics developed in Asia (mainly Japan) are subjected to initial assessment in Japan and in many cases struggle to gain access to markets outside of Asia. Given that the field of antibiotic approval and assessment is small, with few individuals specialized in antibiotics, we were not able to disclose specific agencies and countries and at the same time uphold anonymity of the study participants. While the purpose of this research was not to examine differences between countries, information regarding country setting would have allowed for more in-depth analysis of primarily the challenges and facilitators linked to HTA. Early on it also became clear that participants were not willing to discuss specific antibiotics for the same reason. However, we do not believe that this renders our findings or conclusions invalid. The interviews were conducted over 18 months. Given that much is happening in the regulatory and HTA areas, views could have differed between groups not due to actual differences of opinion but changes in perspectives over time. Additional research is needed to further explore the development and implementation of HTA, for antibiotics, including the implications of current efforts to harmonize the HTA processes in Europe [63]. Finally, exploring the views of the pharmaceutical industry regarding the value of efforts by regulatory authorities and HTA agencies to stimulate antibiotic R&D would be an interesting expansion of research on their role in this domain.

## Conclusions

A shared understanding of limited clinical data and how to deal with this issue is needed amongst stakeholders involved in antibiotic R&D and market introduction to ensure antibiotics reach the market before resistance levels are out of control. Regulatory authorities and HTA agencies, while not considering themselves able to impact antibiotic R&D, could play a more active role in aligning the view of what constitutes an unmet medical need and direct new economic models towards stimulating greater diversity in the antibiotic armamentarium.

## Supplementary Information

**Additional file 1.** Consolidated criteria for reporting qualitative studies (COREQ): 32-item checklist.

**Additional file 2.** Interview guide for semi-structured stakeholder interviews.
