# Supplementary material for: The role of drug regulatory authorities and health technology assessment agencies in shaping incentives for antibiotic R&D: a qualitative study
Source: J Pharm Policy Pract. 2023 Mar 27;16:53. doi: 10.1186/s40545-023-00556-x (PMC10045501; doi:10.1186/s40545-023-00556-x)
Supplement: Supplementary file 2 — Additional file 2. Interview guide for semi-structured stakeholder interviews. [file 40545_2023_556_MOESM2_ESM.docx]

Appendix 2

Interview guide for semi-structured stakeholder interviews

**Basic understanding of the marketing authorization/HTA process with regards to new antibiotics**

1. Which factors are of importance when antibiotics are evaluated?
2. How are these factors ranked in comparison to each other?
   1. Changes over time?
   2. How do regulatory authorities/HTA agencies address AMR?
   3. What are acceptable side-effects for antibiotics?
   4. Has the acceptance for side-effects changed over time?
3. Do the regulatory authorities/HTA agencies differ in their evaluations?

**Changes in the perspective of what an antibiotic is due to AMR**

1. Is AMR affecting our view of what an antibiotic is?
   1. If yes, how are regulatory authorities/HTA agencies dealing with this change?

**Perception of novelty and need. Have any of the antibiotics introduced to market during the past 20 years qualified as novel or filling a public health need?**

1. What do you think the state of the antibiotic R&D pipeline is today?
2. What type of antibiotics would you say we need in the future?
3. How would you define “novel” antibiotic and “public health need”?
4. Would you consider any of the antibiotics that have reached the market during the past 20 years to be novel and/or fill a public health need?
5. How early in the pipeline do you think the value of an antibiotic can be detected?

**Perspective on barriers to getting novel antibiotics to market, both related to regulatory authorities/HTA agencies work of assessing drugs, but also barriers related to other steps of the R&D and market introduction process.**

1. What are the main challenges/barriers to assure that novel and needed antibiotics reach the market?
   1. Specifically in the marketing authorization/HTA process
   2. Apart from the marketing authorization /HTA process
2. Have the regulatory authorities/HTA agencies addressed these issues?
   1. I’ve understood that the requirements for clinical trial data can be reduced if the antibiotic covers an important need. Do you see any problems with this?
   2. How will this impact safety?
3. Do you consider the marketing authorization/HTA process a major barrier for antibiotic R&D?
   1. If yes, in what way?
4. Is there a risk that the pharmaceutical companies terminate antibiotics that could have been of value due to a bad marketing case?

**Perspective on how to improve antibiotic R&D to assure new antibiotics reach the market without risking unnecessary and irresponsible use**

1. Which interventions would, in your opinion, improve the marketing authorization/HTA process for antibiotics in the future?
2. Which incentives are needed to stimulate R&D of antibiotics filling a public health need?
3. Do regulatory authorities/HTA agencies impact the effect of interventions designed to stimulate R&D of new antibiotics?
   1. Should interventions be drug specific or target systems?
   2. What are the costs involved in marketing authorization/HTA process? Can these costs be reduced to make antibiotic R&D more attractive?
4. In the case of drug specific interventions, which characteristics would an antibiotic have to have to qualify for an economic reward?
